# Chronic Diseases in Male Veterans With Multiple Sclerosis

**Published:** 2012-02-09

**Authors:** Sherri L. LaVela, Thomas R. Prohaska, Sylvia Furner, Frances M. Weaver

**Affiliations:** Center for Management of Complex Chronic Care, Hines VA Hospital. Dr LaVela is also affiliated with the Institute for Healthcare Studies, Feinberg School of Medicine, Northwestern University, Chicago, Illinois; University of Illinois at Chicago School of Public Health, Chicago, Illinois; University of Illinois at Chicago School of Public Health, Chicago, Illinois; Center for Management of Complex Chronic Care, Hines VA Hospital, Hines, Illinois, and Program in Health Services Research, Stritch School of Medicine, Loyola University, Maywood, Illinois

## Abstract

**Introduction:**

Chronic disease risk may be high in people with multiple sclerosis (MS). Our objective was to identify chronic health conditions that may disproportionately affect male veterans with MS.

**Methods:**

We collected primary survey data for male veterans with MS (n = 1,142) in 2003 and 2004 and compared the data with 2003 Behavioral Risk Factor Surveillance System secondary data for comparison groups without MS (veteran population, n = 31,500; general population = 68,357). We compared disease prevalence by group and identified variables associated with chronic diseases in male veterans with MS.

**Results:**

Overall, veterans with MS had a high prevalence of hypercholesterolemia (49%), hypertension (47%), diabetes (16%), coronary heart disease (11%), and stroke (7%). Overall and for the subset of people aged 50 years or older, diabetes, hypertension, hypercholesterolemia, coronary heart disease, and stroke were significantly more prevalent among male veterans with MS than among the general population. Diabetes, hypertension, hypercholesterolemia, and stroke were more prevalent overall among male veterans with MS than among the general veteran population; however, except for stroke, differences were not significant for the group aged 50 or older. Explanatory variables (eg, age, education, race) and dynamic associations between conditions (higher odds for each when ≥1 of the other conditions were present) for chronic disease in men with MS were similar to findings in the general population literature for select conditions.

**Conclusion:**

These findings raise awareness of chronic disease in a veteran cohort and help bridge a gap in the literature on chronic disease epidemiology in men with MS. We identified chronic disease priorities that may benefit from focused interventions to reduce disparities.

## Introduction

How idiosyncratic changes related to a person's primary disability, such as multiple sclerosis (MS), affect that person's health and aging process is unclear. Research suggests that people with MS experience more premature illness (1,2), which may result in the presence of more chronic diseases at a younger age, compared with the general population. Factors such as inactivity and immobility may place people with MS at increased risk of developing disabilities, and they may be disproportionately affected by chronic diseases (1,2). Comprehensive research on chronic disease prevalence among people with MS is lacking, especially among men with MS, since MS is more prevalent in women (3).

The Centers for Disease Control and Prevention (CDC) uses the Behavioral Risk Factor Surveillance System (BRFSS) to compile chronic disease prevalence data for the US population, but data specific to people with MS are unavailable. The Veterans Health Administration (VA) is a large health care system that provides care to approximately 28,000 people with MS, a substantial proportion of whom are male (88%) (4). These VA data can provide needed epidemiologic data on chronic disease prevalence in men with MS.

Our objective was to identify chronic diseases that may disproportionately affect male veterans with MS. Diabetes, hypertension, hypercholesterolemia, coronary heart disease (CHD), and stroke were assessed because they are associated with the leading causes of illness and death in the United States (5).

## Methods

### Design

We collected primary data during 2003 and 2004 using a cross-sectional survey mailed to male veterans with MS. We obtained secondary data from the 2003 CDC BRFSS (6) to provide comparison group data for the general veteran population and general population. The institutional review board at the Hines VA Hospital and the Office for the Protection of Research Subjects at the University of Illinois at Chicago approved the study.

We used similarly worded questions from CDC BRFSS modules (6) to design the 65-item Multiple Sclerosis Health Care Questionnaire (MS-HCQ) (survey instrument available on request from the corresponding author) to collect primary data on sociodemographics, health behaviors, and chronic disease prevalence in veterans with MS. We included additional questions to assess MS duration and age at diagnosis.

The BRFSS survey is a standardized instrument used to monitor disease, health, and risk behaviors in the US population (50 states, the District of Columbia, Puerto Rico, Guam, and the US Virgin Islands). BRFSS data are collected annually from a probability sample of households with landline telephones. Trained interviewers conduct surveys by telephone. We obtained data from the 2003 BRFSS (6) for the general veteran population and general population comparison groups. Using formulas established by CDC, we weighted these data for selection probability (additional details may be found at www.cdc.gov/brfss/technical_infodata/weighting.htm).

### Sample

VA researchers mailed surveys to a national cohort of veterans with MS who were members of a congressionally chartered veteran service organization (VSO). The sample included people with an MS diagnosis (confirmed by Veterans Benefits Department for VSO eligibility) who were both current users and nonusers of VA health care. We distributed surveys to 2,940 veterans with MS; 735 were returned undeliverable. A total of 1,305 veterans with MS (163 women, 1,142 men) returned completed surveys, resulting in a 59% (1,305 of 2,205) response rate.

There were 264,684 respondents to the 2003 BRFSS. We excluded data from 160,284 female respondents and 4,543 respondents whose data for veteran status were missing. Therefore, our study sample for comparison groups included 99,857 adult male respondents from the 2003 BRFSS. The general veteran population included respondents who served on active duty in the US Armed Forces and were retired or discharged from military service (n = 31,500); the remaining cohort comprised the male general population (n = 68,357). On the basis of Council of American Survey and Research Organizations guidelines, the 2003 BRFSS had a median response rate of 53% and a median interview completion rate of 77% (7).

### Variables

Proportions of chronic disease prevalence were the main outcome measures, and these were determined by a yes response to questions asking whether they had ever been told by a health professional that they had the disease (diabetes, hypertension, hypercholesterolemia, CHD, or stroke).

Sociodemographic variables were age (continuous), race/ethnicity (self-identified as non-Hispanic white, non-Hispanic black or African American, Hispanic [any race], or other [Asian, Native Hawaiian/other Pacific Islander, American Indian or Alaska Native, or other]), education completed (<12 y, 12 y or equivalent, some college, or college graduate), employment status (employed, unemployed/able to work, unemployed/unable to work, or retired), marital status (married, divorced/separated, widowed, or never married), and geographic region of residence (South, West, Midwest, or Northeast, determined on the basis of US census regions using zip code). Health behavior variables were cigarette smoking (current, past, or never) and chronic drinking (having consumed ≥2 drinks/d in the past 30 days).

### Statistical analyses

We calculated descriptive statistics for age at MS diagnosis (difference in MS diagnosis date and date of birth) and MS duration (difference in MS diagnosis date and survey return date). We conducted bivariate comparisons (*t* tests for continuous and Χ^2^ tests for categorical variables) of male veterans with MS and each of the non-MS male comparison groups overall and for the subset of men aged 50 or older to assess differences between groups. We conducted multivariate analyses for the MS cohort for each chronic disease that resulted in significant bivariate associations between MS and both non-MS groups in the overall comparisons.

We used multivariate analyses to generate odds ratios (ORs) and 95% confidence intervals (CIs) to identify variables associated with the presence of each chronic disease in male veterans with MS. We built separate multivariate logistic regression models for diabetes, hypertension, hypercholesterolemia, and stroke, dichotomizing the dependent variable for each (disease present/not present). Covariates were age, race/ethnicity, age at MS diagnosis, education completed, employment status, marital status, region of residence, smoking and drinking status, and other chronic diseases.

Because of large CDC sample sizes and the potential for significance of small differences, we used random samples to test the significance of bivariate prevalence associations between the MS cohort and each of the CDC comparison groups. Random samples of 1,500 were used for comparisons between the non-MS groups and the MS group (n = 1,142), and random samples of 1,000 were used for the comparisons between the non-MS groups and the MS group (n = 962) for the subset aged 50 or older. To determine the random sample sizes, we calculated power using the Pearson Χ^2^ test of 2 proportions (2-sided test, *P *< .05). We approximated numbers to attain 90% power and to detect a 5% difference in the number of people who had a chronic disease between the MS group and each non-MS group.

We conducted a separate analysis to assess whether the VSO survey respondents with MS were representative of the larger population of veterans with MS. Using VA administrative databases, we captured demographic data for people with MS (according to the *International Classification of Diseases, 9th Revision*, diagnosis code 340) who had used the VA health care system during the data collection period.

Significance was set at *P* < .05. We used SAS version 9.1 (SAS Institute, Inc, Cary, North Carolina) and Stata version 10 software (StataCorp LP, College Station, Texas) for statistical analyses.

## Results

### Participant characteristics

Mean age of MS diagnosis was 37 years, and the average number of years living with MS was 23. In each group, more than half of respondents were married, approximately one-third were college graduates, and the highest proportion of participants were white and resided in the South (Table 1). Compared with veterans with MS and the general veteran population, the general population was younger and a greater proportion was currently employed and never smoked. Chronic drinking was low among all groups, particularly among veterans with MS.

### Chronic diseases

Prevalence of hypercholesterolemia, hypertension, diabetes, and stroke was higher among veterans with MS than among the general veteran and general populations (Table 2). Prevalence of CHD was significantly higher among veterans with MS than among the general population but not among the general veteran population. Among the subset of respondents aged 50 or older, prevalence of stroke was higher among veterans with MS than among the general veteran and general populations. Prevalence of CHD among respondents aged 50 or older was lower among veterans with MS than among the general veteran population and was higher among veterans with MS than among the general population. Prevalence of hypercholesterolemia, hypertension, and diabetes was significantly higher among veterans with MS only compared with the general population.

Multivariate analyses showed that the odds of having diabetes were higher among male veterans with MS who were older, non-Hispanic black or African American (vs white), and past smokers (vs current smokers) and who had hypertension, hypercholesterolemia, or a prior stroke (Table 3). Male veterans with MS who were college graduates had lower odds of having diabetes than their counterparts who did not complete high school. Widowed male veterans with MS were more than 2.5 times as likely to have hypertension as male veterans with MS who were currently married. The odds of having hypertension among male veterans with MS were higher with comorbid diabetes, hypercholesterolemia, prior stroke, and CHD, as well as for chronic drinkers.

Male veterans with MS who were non-Hispanic black or African American (vs white), lived in the Midwest (vs the South), or had a prior stroke had lower odds for hypercholesterolemia. The odds for hypercholesterolemia among male veterans with MS were higher with comorbid diabetes, hypertension, and CHD. Among male veterans with MS, the odds for having a stroke were lower with comorbid hypercholesterolemia but higher with concurrent asthma, CHD, diabetes, and hypertension. Older age at MS diagnosis was marginally associated with a higher risk of stroke.

### Statistical association and representativeness

Presence or absence of significant associations remained across the 5 chronic diseases, with 1 exception: prevalence of diabetes was no longer significant in the MS population compared with the general veteran population (overall). VA administrative data showed comparable characteristics of veterans with MS (vs survey respondents): 86% male (vs 88%), mean age of 58 years (vs 61 y), 85% white (vs 91%), and 65% married (vs 75%).

## Discussion

This study is the first comprehensive national examination of chronic disease prevalence in a large cohort of male veterans with MS compared with population-based CDC surveillance data.

Overall and for respondents aged 50 or older, diabetes, hypertension, hypercholesterolemia, CHD, and stroke were significantly more prevalent among male veterans with MS than among men in the general population. Likewise, diabetes, hypertension, hypercholesterolemia, and stroke were more prevalent, overall, in male veterans with MS than among the general veteran population, but only stroke was significantly more prevalent among the subset of respondents aged 50 or older. Although the higher prevalence of many chronic diseases in the MS cohort relative to the general population was anticipated, this study provided nationally representative estimates of the magnitude of these differences.

### Diabetes

Some studies have reported lower diabetes prevalence among people with MS compared with other cohorts (8); others have reported higher diabetes prevalence among MS cohorts compared with the general population (9), which is consistent with our findings. Higher diabetes prevalence among people with MS may be attributable to muscle disease from nerve demyelination or to adrenocorticotropic hormone and glucocorticoid treatment (9).

We found that male veterans with MS who graduated college were less likely to have diabetes than those with less education, which is supported by reports that men who did not finish high school were more likely to have self-reported (10) and diagnosed (11) diabetes than those who had more education. We found that non-Hispanic black or African American male veterans with MS were nearly 3 times as likely as white male veterans with MS to have diabetes. This finding is similar to that of another study that found that black race was associated with increased odds of diabetes compared with white race but that controlling for education reduced the odds but retained significance (12). In veterans with MS, the disparity in diabetes risk for non-Hispanic black or African American men remained after controlling for education.

Similar to general medical literature, higher odds for diabetes was associated with hypercholesterolemia (13), prior stroke (13), and hypertension (75% of people with type 2 diabetes have hypertension [14]). Much of the illness and death associated with diabetes and its complications can be prevented or delayed by normalizing blood glucose levels, blood pressure, and lipids (15). Some complications that diabetes may lead to, such as vision problems and limb amputation (due to poor circulation) (15), may be more debilitating for people with MS, who often have mobility limitations. Early efforts are needed to screen men with MS who have comorbid conditions that are known risk factors for diabetes, with special focus on non-Hispanic black or African American men and people with less education.

### Hypertension

Male veterans with MS who were chronic drinkers had increased odds of having hypertension, a relationship that has been well documented in the general population (16). Consistent with general medical literature, odds for hypertension in this study were higher with comorbid diabetes (17), CHD (18,19), hyperlipidemia (20), and stroke (21). We found that widowed male veterans with MS were 2.5 times as likely to have hypertension as their counterparts who were married. Becoming widowed may alter peoples' patterns of interaction in the health care system and informal care practices (eg, loss of a person who assists in care and supports self-care management); the associated negative health risks are elevated and long-term for widowed men (22). Not being married has been associated with poor adherence to taking hypertension medication (23). Furthermore, hypertension may be independently associated with an increased risk of ambulatory disability in people with MS (24), making it imperative to address to preserve function in this group.

### Hypercholesterolemia

We found that non-Hispanic black or African American (vs white) race/ethnicity and residing in the Midwest (vs the South) were independently associated with lower odds of hypercholesterolemia in male veterans with MS. Gallup prevalence measures for hypercholesterolemia were comparable for both 2008 and 2009 (eg, white higher than black by ≥5 points and Midwest higher than South by ≥2 points) (25). Consistent with general medical literature, odds of hypercholesterolemia in men with MS were greater with CHD (26), hypertension (20), and diabetes (27). Studies have found different blood cholesterol and lipoprotein levels depending on the phase of clinical stability in which people with MS were (28), which may affect point prevalence values. Further understanding of the higher prevalence of hypercholesterolemia in veterans with MS is warranted, with special attention being paid to its presence in white men and people living in the South.

### Stroke

People with MS may have a higher risk of stroke than people who do not have MS (29). Although the absolute value for stroke prevalence in our study was not alarming, disparities were evident across age groups in male veterans with MS compared with both non-MS groups. Comparable to general medical literature, odds of stroke were higher for people with diabetes (30,31), CHD (30), and hypertension (30). Stroke is a leading cause of death in people with MS (32) and is associated with high hospital use in people with MS (8). Strategies to raise awareness of increased stroke risk and education on stroke warning signs specific to MS (eg, problems with balance/coordination and numbness/weakness may be difficult to distinguish) would be beneficial.

### Limitations

The moderate response rate (59%) for the cohort of veterans with MS may have introduced nonresponse bias. These self-reported data are subject to recall bias. Differences may exist in responses provided by mail (MS-HCQ) versus telephone interview (BRFSS), although comparisons of BRFSS data from the 2 survey modes are "largely equivalent" (33). The comparison groups identified using secondary BRFSS data may have included people with MS, as exclusion was not possible; however, given the small numbers of people with MS relative to the general veteran and general populations (<1%), it is unlikely that chance inclusion of people with MS would have modified any true effect. Data were not available on factors that may influence chronic disease prevalence, such as nutrition, physical activity, genetic risk, or exacerbation periods and MS subtype. Findings from this study may be more generalizable than those of other MS research because the MS cohort is not limited to clinical or patient study samples (which may limit external validity and generate selection bias).

### Conclusion

Overall, diabetes, hypertension, hypercholesterolemia, and stroke were significantly more prevalent among male veterans with MS than among men in both comparison groups. Nearly half of male veterans with MS had hypercholesterolemia and hypertension; these conditions occurring alone or concomitantly are implicated in many other diseases and should be addressed. Although a lower proportion of men were affected by stroke, the prevalence was consistently disparate across age groups. Future studies to examine age at onset and chronic disease severity relative to age- and sex-matched controls are needed to provide knowledge about premature morbidity and aging in people with MS. Further research is needed to understand the effects of clusters of comorbidites in this cohort. Research on the epidemiology of multiple chronic diseases in MS is scarce, and our findings help bridge a literature gap. We identified chronic disease priorities among male veterans with MS that may be targeted for early intervention to improve health and reduce disparities in this population.

## Figures and Tables

**Table 1.** Characteristics of Male Veterans With Multiple Sclerosis Compared With General Veteran and General Populations, United States, 2003-2004^a^^,^ b

Characteristic	Overall					Aged ≥50 Years				

	MS (n = 1,142)	GV (n = 31,500)	*P* c	GP (n = 68,357)	*P* d	MS (n = 962)	GV (n = 25,055)	*P* c	GP (n = 21,316)	*P* d
**Mean age, y**	60.8	59.1	.89	39.5	<.001	63.8	66.0	.79	60.5	.06
**Race/ethnicity^e^**										
Non-Hispanic white	91.0	81.4	<.001	66.6	<.001	92.8	85.6	<.001	73.1	<.001
Non-Hispanic black/African American	5.1	7.6	.002	8.7	<.001	4.2	5.6	.07	8.5	<.001
Hispanic, any race	2.7	5.8	<.001	16.4	<.001	2.1	4.5	<.001	11.5	<.001
Other	1.2	5.2	<.001	8.3	<.001	.9	4.3	<.001	6.9	<.001
**Education completed**										
<12 y	4.1	7.3	<.001	13.8	<.001	4.5	8.5	<.001	17.4	<.001
12 y or equivalent	17.9	31.4	<.001	29.5	<.001	18.6	30.3	<.001	25.5	<.001
Some college	45.3	29.5	<.001	23.8	<.001	43.1	26.9	<.001	18.9	<.001
College graduate	32.7	31.8	.49	32.9	.92	33.8	34.3	.73	38.2	.005
**Employment status^f^**										
Employed	6.2	49.7	<.001	80.2	<.001	4.6	37.7	<.001	57.5	<.001
Unemployed/able	2.2	3.8	.005	7.7	<.001	1.9	2.9	.07	4.9	<.001
Unemployed/unable	40.7	4.9	<.001	4.3	<.001	38.2	4.5	<.001	7.3	<.001
Retired	50.9	41.6	<.001	7.8	<.001	55.2	54.8	.79	30.3	<.001
**Marital status**										
Married	75.5	72.7	.04	57.4	<.001	77.4	76.7	.61	75.1	.11
Divorced/separated	16.8	12.8	<.001	9.1	<.001	15.7	11.0	<.001	12.5	.002
Widowed	3.1	6.4	<.001	1.4	<.001	3.3	8.3	<.001	4.9	.02
Never married	4.6	8.1	<.001	32.1	<.001	3.7	4.0	.61	7.5	<.001
**Geographic region of residence**										
South	29.5	39.7	<.001	36.4	<.001	28.5	38.3	<.001	37.0	<.001
West	22.9	22.1	.54	23.7	.53	23.7	22.2	.26	21.5	.10
Midwest	21.1	20.5	.58	20.7	.75	21.7	21.0	.62	21.3	.77
Northeast	26.5	17.7	<.001	19.2	<.001	26.1	18.5	<.001	20.2	<.001
**Cigarette smoking status**										
Current	16.1	22.8	<.001	25.7	<.001	14.0	17.6	.004	18.9	<.001
Past	63.5	46.1	<.001	22.4	<.001	66.9	53.9	<.001	40.3	<.001
Never	20.4	31.1	<.001	51.9	<.001	19.1	28.6	<.001	40.8	<.001
**Chronic drinker^g^**	2.8	5.6	<.001	7.2	<.001	2.9	4.8	.006	5.1	.003

Abbreviations: MS, veterans with multiple sclerosis; GV, general veteran population; GP, general population.

a Unweighted total sample sizes. All data are reported as weighted percentages unless otherwise indicated.

b Item response for all variables was ≥96%.

c Significance indicated for male veterans with MS vs men in the general veteran population; calculated using Χ^2^ test (except for age, which was calculated using *t* test).

d Significance indicated for male veterans with MS vs men in the general population; calculated using Χ^2^ test (except for age, which was calculated using *t* test).

e Race/ethnicity was self-identified. Hispanic ethnicity included any identified race (or none). Other included Asian, Native Hawaiian/Pacific Islander, American Indian or Alaska Native, or other.

f Employed included employed for wages or self-employed; unemployed was categorized as able to work or unable to work.

g Chronic drinkers were defined as having consumed ≥2 drinks per day in the past 30 days.

**Table 2.** Prevalence of Chronic Diseases Among Male Veterans With Multiple Sclerosis Compared With General Veteran and General Populations, United States, 2003-2004^a^^,^ b

Chronic Illness	Overall					Aged ≥50 Years				

	MS (n = 1,142)	GV (n = 31,500)	*P* c	GP (n = 68,357)	*P* d	MS (n = 962)	GV (n = 25,055)	*P* c	GP (n = 21,316)	*P* d
Coronary heart disease	10.5	11.5	.30	3.3	<.001	12.1	15.0	.01	9.4	.007
Diabetes	15.9	13.6	.02	5.6	<.001	17.8	16.7	.37	14.5	.004
Hypercholesterolemia	48.5	44.6	.008	30.9	<.001	49.7	48.6	.49	44.8	.002
Hypertension	46.7	41.2	<.001	20.9	<.001	49.9	48.1	.26	42.1	<.001
Stroke	7.0	4.2	<.001	1.4	<.001	7.9	5.3	<.001	4.1	<.001

Abbreviations: MS, veterans with multiple sclerosis; GV, general veteran population; GP, general population.

a Unweighted total sample sizes. Data are reported as weighted percentages.

b Item response for all variables was ≥90%.

c Significance indicated for male veterans with MS vs men in the general veteran population; calculated using Χ^2^ test.

d Significance indicated for male veterans with MS vs men in the general population; calculated using using Χ^2^ test.

**Table 3.** Variables Associated With Chronic Diseases in Male Veterans With Multiple Sclerosis, United States, 2003-2004

Variable	Diabetes,^a^ OR (95% CI)	*P*	Hypertension,^a^ OR (95% CI)	*P*	Hyperchol-esterolemia,^a^ OR (95% CI)	*P*	Stroke,^a^ OR (95% CI)	*P*
**Age**	1.03 (1.00-1.05)	.03	1.02 (1.00-1.03)	.06	0.99 (0.97-1.00)	.10	1.03 (0.99-1.07)	.15
**Race/ethnicity**								
Non-Hispanic white	1 [Reference]	NA	1 [Reference]	NA	1 [Reference]	NA	1 [Reference]	NA
Non-Hispanic black/African American	2.78 (1.26-6.12)	.01	0.93 (0.48-1.81)	.83	0.51 (0.26-1.00)	.05	1.04 (0.32-3.32)^b^	.95^b^
Hispanic, any race	1.69 (0.54-5.29)	.37	0.63 (0.25-1.55)	.31	0.63 (0.27-1.47)	.28		
Other	1.88 (0.36-9.90)	.46	1.09 (0.31-3.93)	.89	0.71 (0.21-2.45)	.59		
**Age at MS diagnosis**	1.00 (0.98-1.01)	.59	1.01 (0.99-1.02)	.53	1.00 (0.99-1.02)	.61	1.03 (1.00-1.06)	.07
**Education completed**								
<12 y	1 [Reference]	NA	1 [Reference]	NA	1 [Reference]	NA	1 [Reference]	NA
12 y/equivalent	0.73 (0.29-1.83)	.50	1.34 (0.59-3.05)	.48	1.03 (0.46-2.31)	.95	0.55 (0.11-2.63)	.45
Some college	0.54 (0.23-1.29)	.17	1.08 (0.49-2.38)	.84	1.41 (0.65-3.05)	.39	0.54 (0.13-2.18)	.38
College graduate	0.32 (0.13-0.79)	.01	1.08 (0.49-2.39)	.85	1.33 (0.61-2.90)	.48	0.84 (0.21-3.32)	.80
**Employment status**								
Employed	1 [Reference]	NA	1 [Reference]	NA	1 [Reference]	NA	1 [Reference]	NA
Unemployed/able	1.94 (0.47-8.03)	.36	0.41 (0.13-1.27)	.12	2.01 (0.69-5.88)	.20	NA^c^	NA^c^
Unemployed/unable	1.04 (0.43-2.55)	.92	1.03 (0.58-1.83)	.92	1.10 (0.63-1.94)	.74	NA^c^	NA^c^
Retired	1.02 (0.42-2.52)	.96	1.03 (0.58-1.85)	.91	1.25 (0.70-2.23)	.45	NA^c^	NA^c^
**Marital status**								
Married	1 [Reference]	NA	1 [Reference]	NA	1 [Reference]	NA	1 [Reference]	NA
Divorced/separated	0.82 (0.47-1.44)	.49	0.96 (0.65-1.41)	.83	0.83 (0.56-1.21)	.33	1.17 (0.50-2.70)^b^	.72^b^
Widowed	0.43 (0.12-1.52)	.19	2.54 (1.02-6.33)	.05	2.36 (0.97-5.77)	.06		
Never married	0.15 (0.02-1.15)	.07	0.92 (0.46-1.84)	.81	0.60 (0.30-1.20)	.15		
**Geographic region of residence**								
South	1 [Reference]	NA	1 [Reference]	NA	1 [Reference]	NA	1 [Reference]	NA
West	0.73 (0.42-1.26)	.26	1.02 (0.69-1.49)	.94	0.90 (0.61-1.31)	.57	0.47 (0.18-1.26)	.13
Midwest	0.61 (0.35-1.08)	.09	1.11 (0.75-1.65)	.60	0.57 (0.39-0.85)	.006	0.84 (0.34-2.06)	.69
Northeast	1.07 (0.65-1.74)	.80	0.81 (0.56-1.17)	.27	0.86 (0.60-1.24)	.42	0.55 (0.22-1.41)	.22
**Cigarette smoking status**								
Current	1 [Reference]	NA	1 [Reference]	NA	1 [Reference]	NA	1 [Reference]	NA
Past	1.90 (1.00-3.58)	.05	1.16 (0.77-1.74)	.49	0.78 (0.52-1.17)	.23	0.60 (0.22-1.63)	.31
Never	1.15 (0.54-2.46)	.72	1.15 (0.72-1.86)	.56	0.70 (0.44-1.12)	.14	0.80 (0.27-2.42)	.69
**Chronic drinker**	1.32 (0.40-4.29)	.65	2.65 (1.00-7.03)	.05	1.72 (0.64-4.58)	.28	NA^c^	NA^c^
**Asthma**	1.10 (0.51-2.36)	.81	0.93 (0.53-1.64)	.81	1.14 (0.65-1.99)	.64	5.18 (2.11-12.73)	<.001
**CHD**	1.44 (0.80-2.62)	.23	1.92 (1.13-3.25)	.02	3.17 (1.82-5.52)	<.001	5.78 (2.65-12.61)	<.001
**Diabetes**	NA	NA	2.11 (1.42-3.13)	<.001	2.15 (1.44-3.21)	<.001	3.07 (1.44-6.54)	.004
**Hypertension**	2.15 (1.45-3.19)	<.001	NA	NA	1.81 (1.37-2.40)	<.001	2.38 (1.14-4.95)	.02
**Hyperchol-esterolemia**	2.16 (1.45-3.24)	<.001	1.81 (1.37-2.39)	<.001	NA	NA	0.43 (0.21-0.89)	.02
**Stroke**	3.11 (1.46-6.63)	.003	2.54 (1.19-5.41)	.02	0.43 (0.21-0.89)	.02	NA	NA

Abbreviations: OR, odds ratio; CI, confidence interval; NA, not applicable; MS, multiple sclerosis; CHD, coronary heart disease.

a Sample size for diabetes, hypertension, and hypercholesterolemia was n = 943; stroke, n = 950.

b Combined variable categories for inclusion in stroke regression model due to small cell sizes. For race, combined non-Hispanic black/African American, Hispanic, and other because n <5 for each and for marital status combined never married (n = 2) and widowed (n = 6) with divorced/separated.

c Unable to include employment status and chronic drinker in the stroke regression model due to inadequate cell sizes. (Of persons with stroke, only 2 chronic drinkers and only 2 in the employed reference group).
